# Cognitive emotion regulation for improved mental health: A chain mediation study of Chinese high school students

**DOI:** 10.3389/fpsyg.2022.1041969

**Published:** 2023-01-12

**Authors:** Meijuan Xue, Beile Cong, Yiduo Ye

**Affiliations:** ^1^Psychological Counseling Center, Shanghai Lida University, Shanghai, China; ^2^College of Psychology, Fujian Normal University, Fuzhou, Fujian, China

**Keywords:** family function, cognitive-emotion regulation, psychological capital, life satisfaction, high school students

## Abstract

High school is a critical time for individual development, during which significant physical and mental changes related to puberty occur. Therefore, high school students’ mental health requires more attention from schools, families, and society. Our study explored high school students’ present status and family functioning characteristics, psychological capital, cognitive-emotion regulation, and life satisfaction by surveying 917 students in China. Data were analysed using independent sample *t*-tests, one-way analysis of variance, regression analysis, structural equation modelling, and path analysis. Our results showed that family function was positively correlated with life satisfaction, psychological capital, and positive emotion regulation strategies. Negative emotion regulation strategies were inversely correlated with these variables. The variable of cognitive emotion regulation has two dimensions, *positive* and *negative*. Cognitive-emotional regulation and psychological capital had sequential mediating effects between family function and life satisfaction. The results of this study offer new explanations for the mechanisms of family functioning on life satisfaction, how family functioning affects life satisfaction *via* cognitive-emotional regulation and psychological capital, and have some implications for family parenting. It also provides critical theoretical and practical guidance for schools to emphasise the use of positive cognitive-emotional regulation and the development of students’ psychological capital levels in teaching and learning, thereby improving individual life satisfaction further. These findings highlight the importance of considering emotion regulation strategies and psychological capital when determining students’ life satisfaction, and ensuring a healthy family environment.

## 1. Introduction

Families play a vital role in adolescents’ growth, significantly affecting their physical and mental health and facilitating individual socialisation (Barth, 2017; Shalchi and Esmaeili Shahna, 2018). Bronfenbrenner’s ecological system theory of developmental psychology refers to the interaction between an individual and the environment as a behavioural system. Accordingly, the family acts as an immediate environment in an individual’s life, bridging the relationship between individual development and social adaptation. The social learning theory emphasises that complex behaviours are mainly acquired through observation. Hence, the actions and communication of family members, who serve as role models for learning, parental rearing styles, parent–child relationships, and family structure, will profoundly impact young people (Chi and Ziqing, 2004; Lv, 2019; Huang et al., 2020).

Additionally, the high school period is a critical stage in an individual’s lifespan development. Due to social pressure, the youth tend to face difficulties in their studies and lives. Adolescence is marked by physical and psychological changes, along with an increased frequency of psychological problems. With increased academic pressure regarding the ‘High School Entrance Examination,’ studies have shown that students face issues surrounding peers, parent–child, teacher-student relationships, and other areas (Chen, 2018). Such conflicts can contribute significantly to psychological problems, thereby reducing life satisfaction. A study of 2,861 high school students revealed that 16.79% had severe psychological problems (Zhao et al., 1993).

Effective family functioning helps promote the formation of social abilities and skills, enabling youth to inculcate exemplary habits and experience positive emotions. Conversely, high school students living in an environment with poor family functioning tend to experience more unpleasant emotions. Therefore, this unhealthy environment affects their physical and mental health, interfering with their studies and overall life (Tao et al., 2016). The family functioning theory posits that a family’s failure to achieve basic functions can easily cause various clinical problems for its members. Individuals with poor family functioning experience alienation and psychological dilemmas, such as anxiety and depression (Fang and Sun, 2018). Therefore, students’ mental health is closely related to family factors.

Mental health involves the absence of mental illness and the presence of a positive mental state. A mentally healthy individual is efficient, satisfied, displays a healthy range of emotions, and has excellent social adaptive intelligence. Life satisfaction is a critical indicator of positive mental health (Yang and Ye, 2014) and can be used to measure students’ mental health. In the context of quality education, we need to promote students’ overall development, stimulate their potential and monitor their mental health. Previous studies have shown that family functioning is closely related to life satisfaction (Proctor et al., 2017). When family functioning is better, teenagers’ subjective well-being is enhanced (Herawati and Endah, 2016). Thus, family functioning positively predicts adolescents’ life satisfaction (Oberle et al., 2011). Another study discovered a significant positive correlation between family functioning and the subjective well-being of middle school students (Jia et al., 2018). Therefore, the characteristics and influencing factors of the current life satisfaction of high school students, alongside the relationships between family, school, and the individual, need to be explored. This knowledge will provide a framework of strategies for the mental health development of high school students to enhance their life satisfaction.

Cognitive-emotion regulation strategy refers to an individual’s effort to extract information and adapt to the external environment’s requirements. When faced with an external event, individuals first form a specific understanding of its requirements and then respond accordingly. Researchers have found that cognitive components in individual emotion regulation have a more significant impact on behaviour. Existing studies have shown that individuals can maintain their mental health through an adaptive adjustment of emotions (Luthans et al., 2015). Liu (2009) found that adopting negative cognitive-emotion strategies to deal with negative emotions increased the impact of life events on mental health, whilst positive cognitive-emotion strategies alleviated this impact. The study also investigates the relationship between psychological capital (PsyCap) and family functioning. PsyCap, or psychological energy, refers to the psychological resources that an individual possesses throughout growth and development (Barth, 2017). These emotions primarily encompass hope, optimism, self-efficacy, and resilience. High school is a critical period for the development of PsyCap. According to the conservation-of-resources theory, when an individual lacks resources, they will experience more psychological pressure and negative emotions.

Conversely, when one possesses sufficient resources, their sense of self-worth and ability is strong. Family factors such as family environment, parent–child communication, and parenting styles directly or indirectly impact an individual’s PsyCap. Studies have found that individual self-esteem is low when family functioning is impaired (Shi et al., 2017). Parental rearing patterns can affect adolescents’ life satisfaction through PsyCap (Niwako et al., 2011). Many researchers have confirmed the link between family functioning, individual life satisfaction, and PsyCap. Family functioning processes can directly predict adolescent emotional regulation (Kelada et al., 2018).

Additionally, Lerner (1989) states that personal development is primarily the result of interactions between individuals and key members. Therefore, according to developmental theory, family factors, as well as individual factors, can influence life satisfaction.

Although some studies have revealed the relationship between family functioning and life satisfaction, few have examined the mediating effects of PsyCap and cognitive-emotional regulation on the life satisfaction of high school students. The present study explored the direct relationship between family functioning and the life satisfaction of high school students. Furthermore, it examines the mechanisms of cognitive-emotional regulation and PsyCap in this regard. This study proposes the following research hypotheses: (1) A significant correlation exists between high school students’ family functioning and life satisfaction; (2) Cognitive-emotional regulation plays a mediating role between high school students’ families and life satisfaction; (3) PsyCap mediates between high school students’ family functioning and life satisfaction; (4) Cognitive-emotional regulation and PsyCap The role of cognitive-emotional regulation and PsyCap in mediating the chain between secondary school students’ family functioning and life satisfaction. Factors influencing students’ psychological development and enabling the synergy between family, school, and the individual warrant future family education interventions promoting good student mental health.

## 2. Materials and methods

### 2.1. Participants

This study used cluster sampling to sample 5 schools in Fujian, Hebei, and Shanxi randomly. In total, 1,000 questionnaires were distributed to five Chinese high schools in a class-based unit, and 957 questionnaires were returned, with a recovery rate of 95.7%. Altogether, there were 427 males and 490 females. The mean age was 14.99 years, and the standard deviation was 1.81 years; Incomplete questionnaires or those considered invalid due to extreme response bias were eliminated. The final sample comprised 917 participants, and the response rate was 91.7% (Supplementary Table S1).

### 2.2. Measures

#### 2.2.1. Family function rating scale

The Family Function Rating Scale was compiled by Epstein et al. (1983) according to McMaster’s family function model theory. This study adopted the Chinese version of the scale revised by Li et al. (2013). The scale has 30 test questions across five dimensions: *emotional* (A1–A8) and *active communication* (A9–A13), *egoism* (A14–A19), *problem-solving* (A20–A25), and *family rules* (A26–A30). A four-point Likert scale (1, completely non-conforming to 4: completely conforming) is adopted. The scale involves reverse scoring of some items. The higher the score, the better the family function. The scale’s Cronbach’s α is 0.91. In this study, Cronbach’s α was 0.858, indicating high reliability (see Table 1 for details).

**Table 1:** Scales used in this study.

Name of the scale	Year of publication	Author	Dimensions	Cronbach’s α in this study
Family function rating scale	1983	Epstein et al.	Emotional communication; active communication; egoism; problem-solving; and family rules.	0.858
Adolescent life satisfaction scale	2004	Xing et al.	Friendship; family; school; environment; academic; freedom satisfaction.	0.907
Young students’ PsyCap questionnaire	2015	Ye and Fang	Hope; optimism; self-confidence; resilience.	0.891
Cognitive-emotion regulation questionnaire	2007	Zhu et al.	Negative emotion regulation strategies; positive emotion regulation strategies.	0.805

#### 2.2.2. Adolescent life satisfaction scale

The Youth Life Satisfaction Scale was compiled by Xing et al. (2004) and includes six dimensions: *friendship* (B1, B7, B13, B19, B25, B31, B35), *family* (B2, B8, B14, B20, B26, B32, B36), *school* (B3, B9, B15, B21, B27, B33), *environment* (B4, B10, B16, B22, B28), *academic* (B6, B12, B8, B24, B30, B34), and *freedom satisfaction* (B5, B11, B17, B23, B29). Its seven-point scoring ranges from one (*completely non-conforming*) to seven (*completely conforming*). Items B3, B4, B9, and B10, are reverse-scored questions. Each dimension’s total score, summed, gives the overall life satisfaction score. The scale’s Cronbach’s α is 0.91. In this study, Cronbach’s α was 0.907, indicating high reliability.

#### 2.2.3. Cognitive-emotion regulation questionnaire

This study used the Alblea Cognitive-emotion Regulation Scale, revised by Zhu et al. (2007), having 36 questions and nine dimensions: *self-blame* (D1–D4), *acceptance* (D5–D8), *contemplation* (D9–D12), *positive re-focus* (D13–D16), re-focus plan (D17–D20), *positive re-evaluation* (D21–D24), *rational analysis* (D25–D28), *catastrophising* (D29–D32), and *blaming others* (D33–D36). Among the nine dimensions, self-blaming, contemplation, catastrophising, and blaming others are negative emotion regulation strategies. Active re-focusing, re-focusing on plans, positive re-evaluation, acceptance, and rational analysis are positive. A five-point scoring is used, ranging from one (*never*) to five (*always*). The scale’s Cronbach’s α is 0.81. In this study, Cronbach’s α was 0.805, indicating high reliability.

#### 2.2.4. Young students’ PsyCap questionnaire

This study used the youth PsyCap questionnaire compiled by Ye and Fang (2015). It includes four dimensions: *hope* (C1, C5, C9, C13, C17–C22), *optimism* (C2, C6, C10, C14), *self-confidence* (C3 C7), and *resilience* (C4, C8, C12, C16). Items, C1, C7, C11, and C15 are reverse-scoring questions. A six-point scale is used, ranging from one (*fully non-compliant*) to six (*fully compliant*). A higher score indicates a better student PsyCap. The scale’s Cronbach’s α is 0.905. In this study, Cronbach’s α was 0.891, indicating high reliability.

### 2.3. Research process and statistical analyses

In this study, the test was administered uniformly in a classroom setting. Before questionnaire administration, the subjects were informed of the survey’s significance, purpose, and requirements, and their right to withdraw at any time during the survey. Once informed consent was obtained, the test was administered uniformly by the master testers according to the instructions. The questionnaire was submitted immediately upon completion.

This study used Epidata 3.1 for dual entry and verification of original data; a database was established. The SPSS 22.0 macro, PROCESS, was used to analyse the data. The primary data processing methods were independent sample *t*-tests, one-way analysis of variance (ANOVA), regression analysis, structural equation modelling, and path analysis.

### 2.4. Research ethics and patient consent

This study was approved by the Shanghai Lida University Ethics Committee (Approval Number: SHLDE-2021-02). Verbal informed consent was obtained from all participants. They were informed that participation was voluntary. This study adhered to the latest version of the Ethical Principles of Psychologists and Code of Conduct.

## 3. Results

Whilst distributing and collecting the questionnaires, confidentiality and anonymity were emphasised, and the data collected were limited to scientific research. The participants provided data for all questionnaires. Therefore, any common method deviation sources were reduced by eliminating invalid questionnaires. Harman’s single-factor test was used to assess common method bias. The results revealed 29 factors with characteristic roots greater than one. The variance explanation rate of the first common factor was 14.461%, lower than the critical value of 40%. Therefore, no serious common method deviation existed in this study.

The results showed that participants’ family function, life satisfaction, PsyCap, and, in particular, cognitive-emotion regulation were generally higher than average (Table 2).

**Table 2:** Total variable score.

Dimension	*n*	*Min*	*Max*	*M*	*SD*
Total family function score	917	50	117	92.72	11.46
Total life satisfaction score	917.00	73.00	241.00	165.67	28.84
Total PsyCap score	917.00	31.00	130.00	88.43	15.69
Negative emotion regulation strategies	917.00	23.00	77.00	46.86	7.84
Positive emotion regulation strategies	917.00	34.00	96.00	68.14	8.47

A significant correlation was found between family function, cognitive-emotion regulation, PsyCap, and life satisfaction. Family functioning was positively correlated with life satisfaction, PsyCap, and positive emotion regulation strategies; negative emotion regulation strategies were negatively correlated with family function, life satisfaction, and PsyCap (Table 3).

**Table 3:** Correlation analysis of family function, life satisfaction, PsyCap, and cognitive-emotion regulation.

Variable	Family function	Life satisfaction	PsyCap	Negative emotion regulation strategies	Positive emotion regulation strategies
Family function	1.00				
Life satisfaction	0.501**	1.00			
PsyCap	0.395**	0.678**	1.00		
Negative emotion regulation strategies	−0.199**	−0.225**	−0.285**	1.00	
Positive emotion regulation strategies	0.133**	0.195**	0.304**	0.270**	1.00

**p < 0.01.

First, the mediating effect of PsyCap between family functioning and life satisfaction was assessed using the family function-PsyCap-life satisfaction model (Table 4). The results showed that *c* = 0.501 (*t* = 17.517, *p* < 0.001), *a* = 0.395 (*t* = 13.024, *p* < 0.001), and *b* = 0.569 (*t* = 22.879, *p* < 0.01), indicating that the indirect effect of PsyCap on family function was significant. Additionally, *c’* = 0.276 (*t* = 11.118, *p* < 0.01), indicating that family functioning’s direct effect on life satisfaction was significant.

**Table 4:** Regression analysis of family function, life satisfaction, PsyCap, and cognitive-emotion regulation.

Regression equation		Global fit index		Significance of regression coefficient		
Result variable	Predictor variable	*R*	*R* ^2^	*β*	*F*	*t*
Life satisfaction	Family function	0.501	0.251	0.501***	306.837	17.517
PsyCap	Family function	0.395	0.156	0.395***	169.618	13.024
Life satisfaction	Family function	0.724	0.524	0.276**	502.729	11.118
	PsyCap			0.569**		22.879

***p < 0.001; **p < 0.01.

Further, the mediating effect of the negative emotion regulation strategies between family function and life satisfaction was examined using the family functioning-negative emotion regulation strategies-life satisfaction model (Table 5). The results showed that *c* = 0.501 (*t* = 17.517, *p* < 0.001), *a* = −0.199 (*t* = −6.132, *p* < 0.001), and *b* = −0.131 (*t* = −4.534, *p* < 0.001), indicating that the indirect effect of negative emotion regulation strategies on life satisfaction was significant. Additionally, *c’* = 0.475 (*t* = 16.449, *p* < 0.001), indicating that the direct effect of family functioning on life satisfaction was significant.

**Table 5:** Regression analysis of the relationship between family function, negative emotion regulation strategies, and life satisfaction.

Regression equation		Global fit index		Significance of regression coefficient		
Result variable	Predictor variable	*R*	*R* ^2^	*β*	*F*	*t*
Life satisfaction	Family function	0.501	0.251	0.501***	306.837	17.517
Negative emotion regulation strategies	Family function	0.199	0.039	−0.199***	37.598	−6.132
Life satisfaction	Family function	0.517	0.268	0.475***	166.975	16.449
	Negative emotion regulation strategies			−0.131***		−4.534

***p < 0.001.

Next, the mediating effect of negative emotion regulation strategies between family function and life satisfaction was examined using the family functioning-positive emotion regulation strategies-life satisfaction model (Table 6). The results showed that *c* = 0.501 (*t* = 17.517, *p* < 0.001), *a* = 0.133 (*t* = 4.073, *p* < 0.001), and *b* = 0.131 (*t* = 4.578, *p* < 0.001), indicating that the indirect effect of positive emotion regulation strategies on life satisfaction was significant. Additionally, *c’* = 0.484 (*t* = 16.938, *p* < 0.001), indicating that the direct effect of family function on life satisfaction was significant.

**Table 6:** Regression analysis of the relationship between family function, positive emotion regulation strategies, and life satisfaction.

Regression equation		Global fit index		Significance of regression coefficient		
Result variable	Predictor variable	*R*	*R* ^2^	*β*	*F*	*t*
Life satisfaction	Family function	0.501	0.251	0.501***	306.837	17.517
Positive emotion regulation strategies	Family function	0.133	0.018	0.133***	16.588	4.073
Life satisfaction	Family function	0.518	0.268	0.484***	167.244	16.938
	Positive emotion regulation strategies			0.131***		4.578

***p < 0.001.

To further verify the previous hypothetical medium-chain intermediary model, this study used the bootstrap method to repeat sampling 2,000 times through the SPSS macro PROCESS. Validation model six analysed the chain mediation effect of cognitive-emotion regulation and PsyCap. Direct testing of the mediation effect showed the total indirect effect of positive emotion regulation strategies. PsyCap with a bootstrap 95% confidence interval did not contain any zero values. This result indicated that these two variables had a significant mediating effect between family functioning and life satisfaction. Similarly, regarding the total indirect effects of negative emotion regulation strategies and PsyCap, the bootstrap 95% confidence interval did not contain any zero value. This result indicates that these two variables had a significant mediating effect between family functioning and life satisfaction.

**Figure 1:**
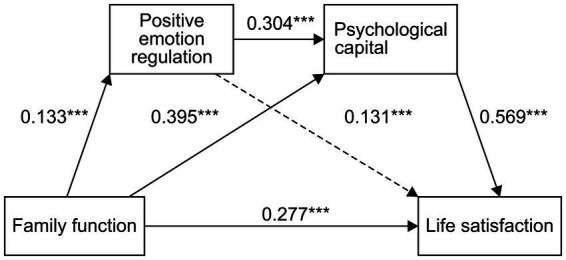
Chain mediation model of family functioning, PsyCap, positive emotion regulation strategy, and life satisfaction. ***is the marked path prominence.

The first mediating effect was composed of three indirect effects. First, the indirect effect 1 produced by family functioning → positive emotion regulation strategies → life satisfaction had its confidence interval including a value of zero, indicating that positive emotion regulation strategies played a role in family functioning and life. No significant indirect effect between family functioning and life satisfaction was detected. Second, indirect effect 2 was produced by family function → PsyCap → life satisfaction. The confidence interval did not include zero, indicating that a significant indirect effect of PsyCap existed between family functioning and life satisfaction (0.2071, 44.86% of the total effect). Third, in the indirect effect 3 produced by family functioning → positive cognitive-emotion regulation strategies → PsyCap → life satisfaction, the confidence interval did not include a value of zero, indicating that the indirect effect produced by this path (0.0195, accounting for 4.59% of the total effect) was significant (Table 7). Figure 1 shows the path diagram from high school students’ family function to life satisfaction.

**Table 7:** Analysis of the mediation effect.

Indirect effect	BootSE	BootLLCI	BootULCI	Relative effect	Percentage
Total indirect effect	0.2246	0.0217	0.1836	0.2689	49.45
Indirect effect 1	−0.0021	0.0037	−0.0100	0.0047	
Indirect effect 2	0.2071	0.0207	0.1672	0.2496	44.86
Indirect effect 3	0.0195	0.0060	0.0087	0.0321	4.59

SE, standard error; LLCI, lower-level confidence interval; ULCI, upper-level confidence interval.

**Figure 2:**
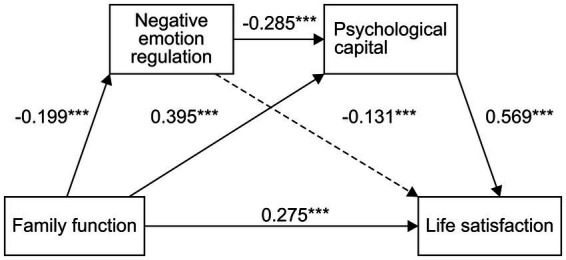
Chain mediation model of family functioning, PsyCap, negative emotion regulation strategy, and life satisfaction. ***is the marked path prominence.

The second mediating effect was composed of three indirect effects. First, in the indirect effect 1 of family functioning → negative emotion regulation strategies → life satisfaction, the confidence interval included a value of zero. This result indicates that negative emotion regulation strategies played a role in family functioning and life satisfaction. No significant indirect effect between family functioning and life satisfaction was detected. Second, indirect effect 2 was produced by family functioning → PsyCap → life satisfaction. The confidence interval did not include zero, indicating that PsyCap had a significant indirect effect between family functioning and life satisfaction (0.1998, accounting for 44.86% of the total effect). Third, for indirect effect 3, produced by family functioning → negative cognitive-emotion regulation strategies → PsyCap → life satisfaction, the confidence interval did not include the zero value, indicating an indirect effect produced by this path (0.0242, accounting for 6.75% of the total effect; Table 8). The path diagram from family functioning of high school students to life satisfaction is shown in Figure 2.

**Table 8:** Analysis of the mediation effect.

Indirect effect	BootSE	BootLLCI	BootULCI	Relative mediating effect	Percentage
Total indirect effect	0.2258	0.0217	0.1848	0.2694	51.61
Indirect effect 1	0.0018	0.0051	−0.0083	0.0119	
Indirect effect 2	0.1998	0.0213	0.1609	0.2435	44.86
Indirect effect 3	0.0242	0.0055	0.0142	0.0359	6.75

SE, standard error; LLCI, lower-level confidence interval; ULCI, upper-level confidence interval.

## 4. Discussion

Our findings showed that high school students’ family functioning and life satisfaction were significantly positively correlated. Further regression analysis found that family function had a significant positive predictive effect on life satisfaction. This result is consistent with Hong et al. (2016) and Ye (2014). Based on these results, we suggest that family is a child’s first classroom. Parents act as the first teachers contributing towards their growth. In healthy functioning families, the members are more inclined to face life with a positive attitude and gain experience whilst solving problems. These strategies improve life satisfaction and promote the development of good mental health.

A significant positive correlation was found between family functioning and the PsyCap of high school students, with family functioning predicting PsyCap. Studies have confirmed that family plays a vital role in psychological development and socialisation (Barth, 2017; Shalchi and Esmaeili Shahna, 2018; White et al., 2018). This finding is consistent with Ji et al. (2018). In other words, better family functioning results in higher PsyCap scores. Individuals with good family function will have better mental health. When high school students face challenges, their families with better family functioning will guide them appropriately, improving their problem-solving ability with subsequent gains in PsyCap.

The family functioning of high school students was significantly negatively and positively correlated with negative and positive emotion regulation strategies, respectively. Further regression analysis found that family functioning could negatively and positively predict negative and positive emotion regulation strategies, respectively. Whilst adolescents’ cognitive-emotion regulation strategies are influenced by many factors, family functioning is critically important (Wang, 2011). Parents specifically act as role models for their children. In well-functioning families, parents are more sensitive to their children’s emotional responses, and they often interact with their children. Communication further promotes the formation of children’s positive emotion regulation strategies. Individuals with inadequate family functioning are treated more poorly by their parents. Their parents’ evaluation further affects their self-evaluation, adversely influencing their cognitive-emotion regulation strategies.

The PsyCap of high school students was significantly negatively and positively correlated with inadequate and effective emotion regulation strategies, respectively. Further regression analysis found that PsyCap could positively and negatively predict healthy and impaired emotion regulation strategies, respectively. Therefore, individuals with high PsyCap should have increased mental energy, thus adopting more constructive cognitive-emotion regulation strategies when facing life events and confronting problems with an optimistic attitude.

The PsyCap of high school students was significantly positively correlated with life satisfaction. Further regression analysis revealed that PsyCap could predict life satisfaction. This finding is consistent with Wang (2014) and Wang (2012), who concluded that family functioning and PsyCap were significantly positively correlated. Thus, the better the individual’s family functioning, the higher the level of PsyCap. The healthier the family functioning, the more constructive high school students’ experiences are with their families. This situation enables them to learn effective problem-solving, communication, and adaptation to unpleasant emotions or unfavourable life events. Healthy family functioning is conducive to improving junior high school students’ subjective evaluation, thereby increasing life satisfaction.

High school students’ unhealthy or constructive emotion regulation strategies were significantly negatively and positively correlated with life satisfaction, respectively. Further regression analysis found that positive and negative cognitive-emotion regulation strategies could positively and negatively predict life satisfaction. Individuals who adopt constructive emotion regulation strategies are in a healthy mindset when they encounter problems or challenging life events, adopting rational analysis, positive re-evaluation, and refocusing on planning, acceptance, and rationalisation. However, individuals who adopt unhealthy cognitive-emotion regulation strategies adopt catastrophic thinking, blame others, engage in self-blaming, and ruminate when encountering problems. When looking at problems pessimistically, individuals are more likely to fall into an impaired emotional state. When they vent their emotions, they are more likely to engage in harmful behaviours. These actions damage interpersonal relationships. The continuous accumulation of painful emotions is difficult to manage. Without a change in attitude, high school students’ cognition will be slow to mature, and their emotional experience will be diminished.

The present study discovered that family functioning influences life satisfaction *via* cognitive emotion regulation strategies and PsyCap when these two mediating variables are introduced as mediating variables. Previous research has primarily focused on family functioning, PsyCap, and life satisfaction. Few researchers have investigated cognitive-emotional regulation’s role in these areas. The current study discovered a novel relationship between these four factors, particularly the role of cognitive-emotional regulation in them, adding to the theory of family functioning from the perspective of cultural differences. The Chinese culture emphasises ‘filial piety’ and the idea that ‘filial piety is the first of all virtues,’ Thus, the family has a profound and lasting influence on the individual’s growth and development. Simultaneously, this study reveals the influence of family on individual life satisfaction and family functioning’s influence *via* cognitive-emotional regulation and PsyCap, and the relationship between them, which has some guiding significance for family parenting in the Chinese cultural context. From the perspective of school education, active roles played by family and school are crucial. This finding suggests that future education and teaching work should improve students’ attribution strategies and teach constructive cognitive-emotion regulation, and raise PsyCap levels. Whilst school education is important for influencing life satisfaction, we must also consider the impact of family education on individuals.

### 4.1. Research significance

#### 4.1.1. Theoretical implications

The findings of this study add to the theory of family function by providing a new explanation for the mechanism of family function on life satisfaction.

#### 4.1.2. Practical implications

Family parenting can benefit from understanding how family functions affect life satisfaction through cognitive emotion regulation and PsyCap. Simultaneously, schools must teach students accurate attributions and constructive cognitive-emotional regulation to increase PsyCap and promote beneficial psychological qualities such as self-confidence, hope, optimism, and resilience in future education and teaching. Finally, schools should also use the combined efforts of the school, family, and society to influence students. This line of thought provides essential theoretical and practical guidance.

### 4.2. Strengths and limitations

This study found that the chain mediation effect of cognitive-emotion regulation enriched the relationship between family function and life satisfaction. However, the following shortcomings exist: (1) The use of variables in this study has limitations, and the variables can be expanded in the follow-up study based on the content of this study. (2) The study’s population is students, but it can be expanded to adults in the follow-up study, which could lead to new findings. (3) Cross-cultural limitations and differences may exist in the Chinese context, which focuses more on family culture.

However, the information was gathered through a questionnaire survey. Experimental and interview methods can be used to supplement future research content.

### 4.3. Relevance to the practise of school psychology

This study identified the essential mediating role of cognitive-emotional regulation strategies and PsyCap. In addition, based on the research findings combined with real-life contexts, this study concludes that school psychologists must focus on improving the accuracy of students’ attributions. They should encourage students to adopt reasonable cognitive-emotional regulation strategies to increase their PsyCap. Furthermore, school psychologists could play a pivotal role in the combined efforts of school, family, and society to influence students. Finally, the role of family education in individual development is emphasised.

## 5. Conclusion

Our study examined the relationship among high school students’ family functioning, PsyCap, cognitive-emotion regulation, and life satisfaction. It found that practical methods of improving life satisfaction and implementing strategies for effective mental health development are long-term challenges that must be addressed.

## Data availability statement

The original contributions presented in the study are included in the article/Supplementary material, further inquiries can be directed to the corresponding author.

## Ethics statement

The studies involving human participants were reviewed and approved by Shanghai Lida University Ethics Committee. The patients/participants provided their written informed consent to participate in this study.

## Author contributions

MX: conceptualisation, methodology, software, validation. BC: visualisation, investigation. YY: reviewing and editing. All authors contributed to the article and approved the submitted version.

## Conflict of interest

The authors declare that the research was conducted in the absence of any commercial or financial relationships that could be construed as a potential conflict of interest.

## Publisher’s note

All claims expressed in this article are solely those of the authors and do not necessarily represent those of their affiliated organizations, or those of the publisher, the editors and the reviewers. Any product that may be evaluated in this article, or claim that may be made by its manufacturer, is not guaranteed or endorsed by the publisher.
