# Supplementary material for: Cognitive emotion regulation for improved mental health: A chain mediation study of Chinese high school students
Source: Front Psychol. 2023 Jan 12;13:1041969. doi: 10.3389/fpsyg.2022.1041969 (PMC9878322; doi:10.3389/fpsyg.2022.1041969)
Supplement: Supplementary file 1 [file Table_1.DOCX]

Supplementary Material

**Supplementary Table S1.** Participants’ information

| Variable | Category | Number of people | Percentage (%) | Effective percentage (%) | Cumulative percentage (%) |
| --- | --- | --- | --- | --- | --- |
| Sex | Male | 427 | 46.6 | 46.6 | 46.6 |
|  | Female | 490 | 53.4 | 53.4 | 100 |
| Grade | First grade of junior high school | 203 | 22.1 | 22.1 | 22.1 |
|  | Second grade of junior high school | 100 | 10.9 | 10.9 | 33 |
|  | Third grade of junior high school | 194 | 21.2 | 21.2 | 54.2 |
|  | First grade of high school | 263 | 28.7 | 28.7 | 82.9 |
|  | Second grade of high school | 157 | 17.1 | 17.1 | 100 |
| Whether you are an only child | Yes | 163 | 17.8 | 17.8 | 17.8 |
|  | No | 754 | 82.2 | 82.2 | 100 |
| Family residence | Town | 446 | 48.6 | 48.6 | 48.6 |
|  | Countryside | 471 | 51.4 | 51.4 | 100 |
| Father’s education level | Junior high school and below | 614 | 67 | 67 | 67 |
|  | High school or technical secondary school | 242 | 26.4 | 26.4 | 93.3 |
|  | bachelor’s degree and above | 61 | 6.7 | 6.7 | 100 |
| Mother’s education level | Junior high school and below | 661 | 72.1 | 72.1 | 72.1 |
|  | High school or technical secondary school | 203 | 22.1 | 22.1 | 94.2 |
|  | Junior college or bachelor’s degree and above | 53 | 5.8 | 5.8 | 100 |
| Family economic status | Well | 151 | 16.5 | 16.5 | 16.5 |
|  | General | 645 | 70.3 | 70.3 | 86.8 |
|  | Poor | 121 | 13.2 | 13.2 | 100 |

Average age by grade: junior: 13–15-year-olds; senior: 16-year-olds; sophomore: 17-year-olds
